# Few-Shot Learning-Based Light-Weight WDCNN Model for Bearing Fault Diagnosis in Siamese Network

**DOI:** 10.3390/s23146587

**Published:** 2023-07-21

**Authors:** Daehwan Lee, Jongpil Jeong

**Affiliations:** Department of Smart Factory Convergence, Sungkyunkwan University, 2066 Seobu-ro, Jangan-gu, Suwon 16419, Republic of Korea; dhlee0915@g.skku.edu

**Keywords:** few-shot learning, Siamese Network, WDCNN (Deep Convolutional Neural Networks with Wide First-layer Kernels), Depthwise Separable Convolution layer, bearing fault diagnosis

## Abstract

In this study, bearing fault diagnosis is performed with a small amount of data through few-shot learning. Recently, a fault diagnosis method based on deep learning has achieved promising results. Most studies required numerous training samples for fault diagnosis. However, at manufacturing sites, it is impossible to have enough training samples to represent all fault types under all operating conditions. In addition, most studies consider only accuracy, and models are complex and computationally expensive. Research that only considers accuracy is inefficient since manufacturing sites change rapidly. Therefore, in this study, we propose a few-shot learning model that can effectively learn with small data. In addition, a Depthwise Separable Convolution layer that can effectively reduce parameters is used together. In order to find an efficient model, the optimal hyperparameters were found by adjusting the number of blocks and hyperparameters, and by using a Depthwise Separable Convolution layer for the optimal hyperparameters, it showed higher accuracy and fewer parameters than the existing model.

## 1. Introduction

Manufacturing competitiveness is a critical factor in the era of global competition and the Fourth Industrial Revolution. In addition, the share of manufacturing in Korea’s GDP is high compared to other countries. To secure manufacturing competitiveness, product quality and facility management are important. It is difficult to manage production facilities on-site, and most companies, except large domestic companies, do not have personnel to maintain facilities. In many cases, production is suspended until repair personnel arrive. This results in various losses such as static losses due to equipment fault and downtime due to equipment fault repair time, making it difficult to produce stable products. The limited environment and limited data available on a real manufacturing floor require utilization of effective algorithms. In previous research on fault diagnosis, SVM (support vector machine) [1], Random Forest [2], wavelet packet [2] and K-nearest neighbor [3] were used for feature design, selection and reduction. In addition, X. Jianhui et al. proposed principal component analysis (PCA) [4] and applied it to high-dimensional unbalanced fault diagnosis data. However, the learning speed is slow and it takes a long time to find the optimized model, which makes it difficult to diagnose large amounts of data. In addition, due to the various bearing operating environments, it is difficult to extract the fault characteristics of the bearing. For these reasons, research on bearing failure diagnosis still needs to be improved. In recent years, many deep learning research methods have been developed for fault diagnosis. Unlike traditional intelligent models, deep learning can automatically learn a high-level representation of the input data through multiple nonlinear layers, avoiding signal processing and heavy manual feature extraction. Previous bearing fault studies utilizing deep learning include CNNs (Convolutional Neural Network) [5,6], RNNs (Recurrent Neural Network) [7,8,9], and auto-encoders [10,11]. Other studies include GANs (Generative Adversarial Network) [12,13]. Signal translation and time stretching using limited data, and data augmentation using GAN [14].

In the GAN’s method, first, the original vibration signal is preprocessed by applying a fast Fourier transform to obtain the frequency spectrum of the defect sample. Second, the spectral data are used as the input to the GAN to generate synthetic prime samples according to the data distribution of the actual samples. Other GAN methods have proposed categorical adversarial autoencoders for unsupervised fault diagnosis. However, the quality of sample data generated by GANs decreases with the quality and quantity of real data when generating auxiliary data, resulting in poor classification accuracy when training the algorithm. Therefore, the effectiveness of this method is highly dependent on the quality and quantity of real data [15,16,17].

In the above studies, many data-driven and deep learning-based techniques were applied to improve accuracy, but most require a large amount of training data such as vibration, sound, motor, and current signals. However, it is difficult to obtain enough quality data samples to train all the fault type classifications in real manufacturing sites. Two problems can be found in previous studies. Bearing fault diagnosis research is still conducted in a limited environment.

First, collecting a sufficient amount of data for each bearing failure condition is costly and time-consuming because most degradation occurs slowly over time. Second, in actual manufacturing, the probability for occurrence of bearing fault types varies depending on the production conditions, and it is difficult to collect enough samples for each failure type due to the imbalance of working conditions and failure types. For this reason, the amount of fault data is very small. Also, the sampled data are increasingly complex and diversified, with many different defect categories, making it difficult to label all defects. In addition, defect categorization requires specialized knowledge of the defect. Therefore, most sampled data from the production floor are unlabeled. Moreover, as equipment ages, it becomes more susceptible to failures and difficult to maintain, creating a need for effective algorithms to improve productivity, availability, and safety [18,19].

Second, the number of parameters increased as the model became more complex and computationally expensive. CNNs have evolved to become more layered to achieve high accuracy [20]. The deepening of CNNs has greatly increased the computation and memory usage, resulting in very poor inference speed in low-performance environments such as in the case of mobile equipment. To solve this problem, a CNN model with an improved structure has been proposed to minimize the loss of accuracy while reducing the amount of computation in mobile environments such as MobileNet. Along with these advances in CNNs, most of the previous studies in equipment fault diagnosis considered only accuracy improvement, and hybrid CNN models that combine several models such as CNN [21], LSTM (Long Short-Term Memory) [22], CNN and LSTM [23], and CNN and attention module transformer [24] were also used. Most previous bearing fault diagnosis studies have compared models based on accuracy alone. In a number of Case Western Reserve University (CWRU) papers, most of the models presented by the authors achieved greater than 95% accuracy. However, most of these high-accuracy models had many parameters. Despite the model accuracy, it is difficult to apply it to a fast-moving, complex production floor if it is time-consuming due to the large number of parameters. In addition, most approaches continuously increase the parameter or width of the model, which leads to model complexity and a large number of parameters. This increases the demands on computer hardware, the level of operational equipment, and the difficulty of intelligent fault diagnosis.

Few-shot learning, which allows for learning with small amount of data, was first discussed in the 1980s and has made great strides in addressing the problem of sparse data [25]. Koch et al. [26] proposed a Siamese neural network for image recognition. Zhang et al. [27] created a CNN-based few-shot learning model for bearing fault diagnosis. Nanging Dong [28] addressed the problem of domain adaptation in one-shot image classification by combining domain adaptation and one-shot learning. Snell et al. [29] proposed a prototype networks. O. Vinyals [30] adopted the ideas of matrix learning based on deep neural networks and recent advances in augmenting neural networks with external memory to apply matching networks for one-shot learning.

In this study, we identify the optimal parameters and accuracy for the WDCNN model that achieved high accuracy along with few-shot learning that can learn efficiently with small data. To reduce parameters more effectively while maintaining maximum accuracy, we use a Depthwise Separable Convolution layer, one of the convolution operations. In this paper, we proposed a method for finding an efficient bearing defect diagnosis model based on few-shot learning in a Siamese Network with limited training data.

The contributions of this paper are as follows. First, we propose a WDCNN Siamese Network Architecture based on a few-shot learning model. This architecture consists of three stages and utilizes two types of change targets (block, hyperparameter) and a Depthwise Separable Convolution layer to determine the accuracy of parameter change according to the change target. First, we assessed the accuracy and parameter change by the number of blocks. In this experiment, we used not only a regular convolution layer but also a Depthwise Separable Convolution layer. First, the parameters for each number of blocks of WDCNN composed of convolution layers decreased from 103,993 to 51,449, and the accuracy varied by 82%∼99% for each sample.

Second, we used a Depthwise Separable Convolution layer in addition to the convolution layer in the existing WDCNN to reduce parameters more efficiently. Depthwise Separable Convolution is one of the computational techniques of CNN, which is a computational method that performs Depthwise convolution and pointwise convolution. After selecting efficient hyperparameter combinations in the block number adjustment and hyperparameter adjustment methods presented in this paper, we used a Depthwise Separable Convolution layer to reduce the number of parameters to 32,089, which is about 20,000 less than the existing baseline model (WDCNN block5) [26], which achieved higher accuracy than the base model in most samples.

Table 1 shows the comparison between previous works and proposed methods.

This paper is organized as follows. Section 2 introduces bearing, few-shot learning, Siamese Network, CNN and Depthwise Separable Convolution. Section 3 describes the proposed few-shot learning model based on WDCNN Siamese Network architecture in each step. Section 4 describes the experimental environment, datasets, evaluation measures, and results of the three experiments. Finally, Section 5 presents the conclusions of the three experiments and future research directions.

## 2. Related Works

### 2.1. Few-Shot Learning

**Figure 1:**
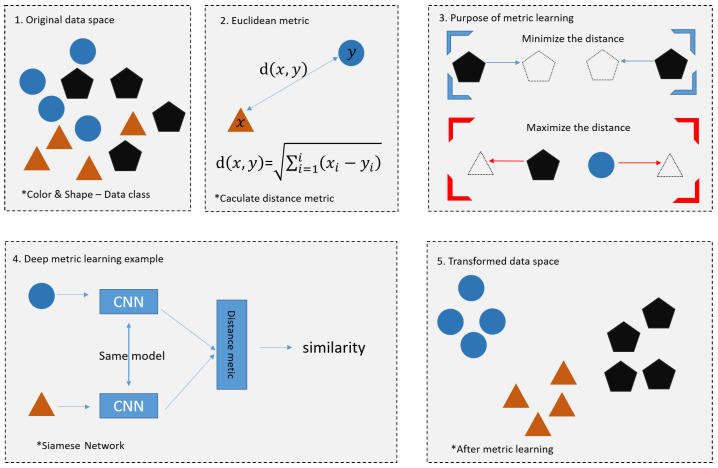
Methods based on metric.

Few-shot learning was first addressed in the 1980s and is a type of meta-learning [32,33]. In recent years, few-shot learning has made great progress in solving the problem of data sparsity [34,35]. It is a learning method that focuses on learning effectively with only a small amount of data in the absence of a large amount of training data and is a type of meta-learning. In general, while supervised learning in classification models is about predicting the correct class for a given set of input data, the core of supervised learning is about learning how similar or different a given set of data is from other sets of data. There are two main types of few-shot Learning methods: the data-driven approach and model-based approach. The data-driven approach is a method to generate enough data to train a model by applying a transformation or using a Generative Adversarial Network (GAN). However, GAN has a clear limitation in that the quality of the originally generated data is low and the quality of the secondary data generated is low if the amount is small. In addition, it has large time and memory requirements, and the data in the support set cannot guarantee the population of the data. The Model-based Approach method is to learn the similarity between feature vectors so that the model can distinguish between images of the same class and images of different classes, or to introduce regularization to prevent the model from overfitting with a small amount of data. Model-based approaches include metric-based approaches that use metrics such as distance or similarity and methods that utilize graph neural networks, which have received great attention recently [36]. Figure 1 shows the methods based on metric.

Metric learning is a methodology that uses a deep learning model to learn an embedding function that quantifies the similarity among data points. It uses Euclidean metric and Siamese Network to calculate the distance between data through similarity. The square labeled 1. represents the original data space, and 2. represents the Euclidean metric. Euclidean metric is a formula for finding the distance between two points in n-dimensional space. The square labeled 3. for the purpose of matrix learning, closes the distance between similar figures and increases the distance between different ones.

**Figure 2:**
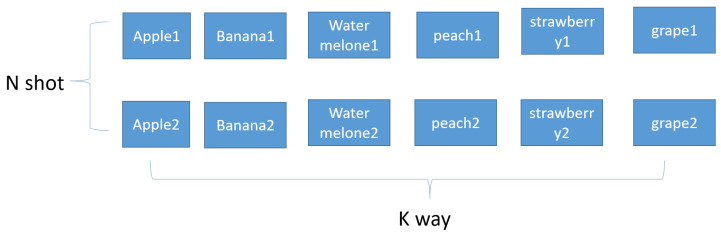
Supportset.

The last picture is the picture after metric learning is performed, and it can be seen that the distance between the same figures is close, and the distance between different figures is long. Few-shot learning is different from the dataset of existing deep learning and is divided into training set/support set/query set. Figure 2 shows the Supportset.

The support set can be represented as a k-way n-shot. The difficulty level depends on the composition of the support set. K-way refers to the number of classes in the support set. For example, if K is 6 (ex: 6-way), the support set has six classes. N-shot is the number of samples in each class. In the example above, assuming a 6-way 2-shot, each of the 6 classes has two samples. In general, the higher the number of shots, the higher the accuracy, while the lower the number of shots, the lower the accuracy. On the other hand, accuracy decreases when the number of ways increases, and the accuracy increases when the number of ways decreases. In general, five ways, one shot, three shots, and five shots are used in a few-shot run. Few-shot learning trains a model on a data training set. The goal of training is to learn the similarities and differences between the given data objects. The model is trained to recognize the similarities and differences between the input data. Therefore, the trained model can recognize whether the content between two objects is the same or different. After training, with the additional information provided by the support set, the trained model compares the support set and the query set to find the data in the support set that are most similar to the query set.

### 2.2. Siamese Network

**Figure 3:**
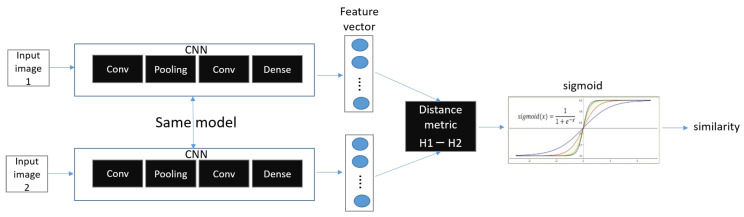
Binary of Siamese Network.

A Siamese neural network (pronounced “Siamese miss”) is a deep neural network that uses two identical or different input data, sharing the same network structure and the same parameters (weights), to perform a comparison operation on each of the output vectors. The Siamese nets structure was first introduced in the early 1990s. Bromley, Jane used Siamese nets to solve signature verification as an image matching problem [37]. In addition, the Siamese net structure itself was introduced in 2005 by Prof. Yann LeCun’s group. They present a method for training similarity metrics on data. This method can be used for recognition or verification applications where the number of categories is very large and unknown during training and the number of training samples for a single category is very small [38]. In addition to the Siamese Network, there is also the Matching Network and the Prototype Network [39]. Siamese Networks are used in conjunction with binary loss or triplet loss. Figure 3 shows the binary of Siamese Network.

**Figure 4:**
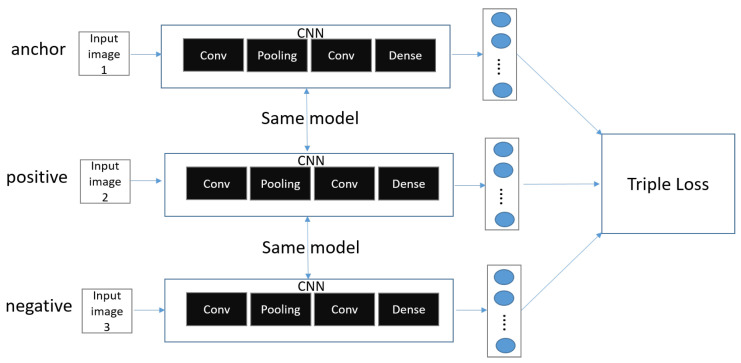
Triple of Siamese Network.

Figure 4 shows the triple of Siamese network.

Unlike regular CNNs, in binary loss, there are two CNN networks, both with the same parameters and network structure. Therefore, they are also called twin networks. Siamese Networks usually use the CNN model, but other models can be used as well. A Siamese Network receives two inputs of data (ex: image). The neural network outputs two feature vectors extracted from the two input data and calculates a vector of values resulting from the difference between the two feature vectors (z = |h1 − h2|), called h1 and h2, respectively. As a result, we use multiple dense layers to process the difference between the vectors and finally apply a sigmoid activation function to obtain a number between scalars 0 and 1. The output is close to 1 (positive samples) if the two images are of the same class, and 0 (Negative Samples) if they are of different classes.

### 2.3. CNN

**Figure 5:**
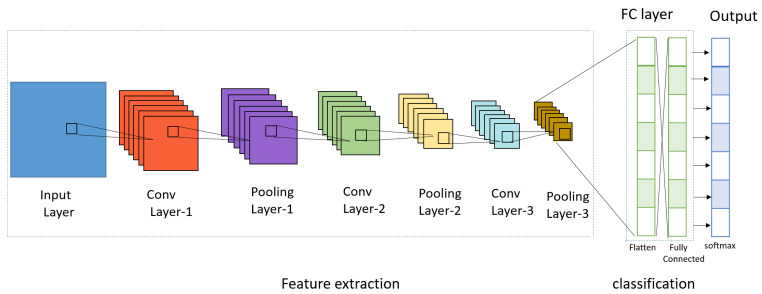
CNN.

CNN, which is defined as the convolution neural network, is a deep learning model inspired by the structure of biological vision systems. It is a method in which the problems encountered when processing data such as images or videos in a regular deep neural network are complemented by a preprocessing operation called convolution. CNN solves the problems of general DNNs, and CNN models are divided into 1D, 2D, and 3D, but general CNNs usually refer to 2D, which is used for image classification. Here, D stands for dimensional, and depending on the type of input data, 1D, 2D, and 3D CNN models are used. In general, 1D refers to time series data, 2D refers to horizontal × vertical black and white images, general CNNs basically use one-dimensional data, and 3D refers to horizontal × vertical × channel (color) color images. A CNN uses the image as raw input and builds a hierarchy of features while retaining spatial and local information. The key point of CNNs is to look at parts of an image rather than the whole and to recognize how a pixel in an image relates to its neighbors. However, when an image is the input, conversion of the image to one dimension (e.g., flattening line data) loses the spatial/topological information of the input, making it more difficult to find a specific object in the entire picture. Figure 5 shows the CNN.

**Figure 6:**
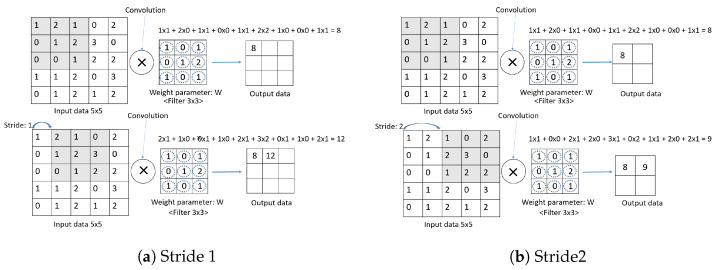
Compute convolution as stride size changes (**a**) Stride 1 (**b**) Stride 2.

The main structure of CNN consists of three parts: the convolution layer, pooling layer, and the fully connected layer (FC Layer). In addition, the convolution layer and max pooling layer are called the feature extraction part, which is repeatedly stacked, and the fully connected layer is divided into the classification part, which constitutes the fully connected layer and applies softmax to the last output layer. The Convolution layer is a required element that reflects the activation function after applying a filter to the input data. The Pooling layer is an optional element that is used to reduce the exponential increase in computation and emphasize certain features. The fully connected layer consists of a flatten layer and a softmax layer. The flatten layer changes the data type to a fully connected network, and the softmax layer performs classification. Convolution is also called convolution in English. It is an m × n-sized matrix called a kernel or filter that overlaps an image (ex: height × width) from top to bottom. The matrix multiplies the values of each image in the m × n-sized overlap with the values of the elements in the kernel and adds them all together as an output. In this case, the image is traversed sequentially according to the size of stride, from the top left corner to the bottom right corner. Figure 6 shows the convolution product operation as a function of stride size. A convolution is a type of product of two functions. The shaded area represents the range of the kernel and input data to be calculated.

Stride is the interval at which the filter is traversed. The convolution operation is performed by moving the filter (kernel) one space for each input data item. In Figure 6a, the output for a stride of 1 is 3 × 3. In Comparison, in Figure 6b, the output at Stride 2 is 2 × 2. The output is smaller when the stride is larger than when it is smaller.

**Figure 7:**
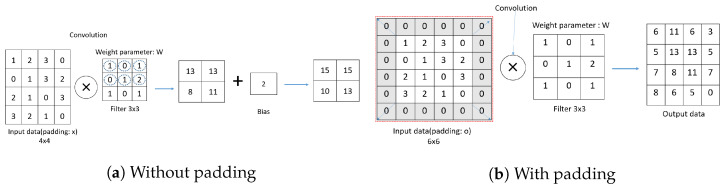
Compute convolution as stride size changes (**a**) Without Padding (**b**) With Padding.

Figure 7 shows the padding. Padding means filling in certain values around the data before the convolution product operation.

**Figure 8:**
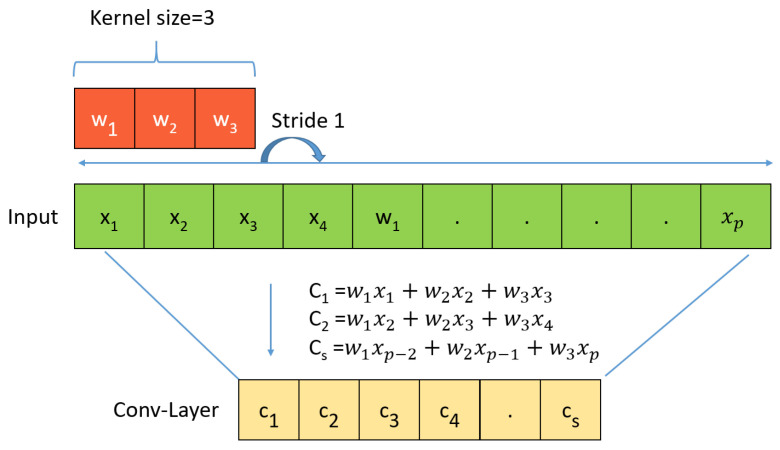
1D CNN.

Figure 7a shows the data without padding, and Figure 7b includes 0 padding, and the shaded area represents the added padding 0 area. There is a difference between the results with and without padding. Figure 6 and Figure 7 show that increasing the stride reduces the size of the output data, and applying padding increases the size of the output data. Figure 8 shows the 1D convolution computation process.

The 1D CNN applied in this study is suitable for natural language processing and analyzing of sensor and signal data. It is also useful for identifying simple patterns in data and is well suited for analyzing time sequences.

Figure 8 $C_{1}$ is the product of the filter (kernel) and input data, as shown in Equation (1). Unlike 2D convolution, the 1D convolution operation is performed in the horizontal direction. Then, with stride 1, we move it one space horizontally and calculate the new product of the filter (kernel) and input data, C${}_{2}$, as shown in Equation (2). Continuing the expression, an expression such as $C_{s}$ is obtained.

(1) $$C_{1}=w_{1}x_{1}+w_{2}x_{2}+w_{3}x_{3}$$

(2) $$C_{2}=w_{1}x_{2}+w_{2}x_{3}+w_{3}x_{4}$$

(3) $$C_{s}=w_{1}x_{p}-2+w_{2}x_{p}-1+w_{3}x_{p}$$

Figure 8 shows an array of length P with P elements (input data) and a filter of length 3 (kernel). WDCNN is Deep Convolutional Neural Networks with Wide First-layer Kernels, which uses a large kernel size for the first CNN layer and uses more layers than the CNN used in the existing method to create a deep model. If the kernel of the first layer is made small, it is easily disturbed by the high-frequency noise common in industrial environments. Therefore, to capture useful information of the vibration signal in the middle and low frequency bands, we first extract the features using a broad kernel.

### 2.4. Depthwise Separable Convolution

**Figure 9:**
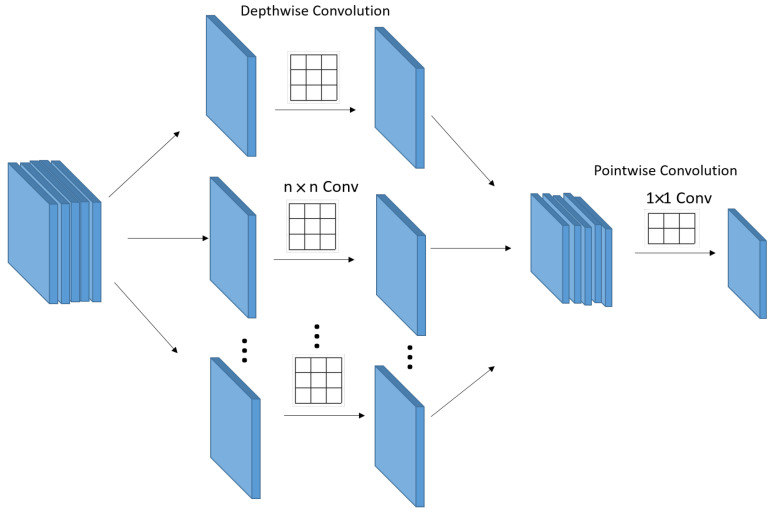
Depthwise Separable Convolution.

In general, to scale up the receptive field in a CNN, we can think of scaling up the kernel size or stacking many convolution layers. However, both of these methods can be inappropriate because they significantly increase the amount of computation. Therefore, various convolution techniques have emerged in deep learning to lighten the computational load while extracting only the most significant information without losing information. Convolution, Separable convolution, Depthwise convolution, Depthwise Separable Convolution, and pointwise convolution are representative. Figure 9 shows the Depthwise Separable Convolution.

Depthwise Separable Convolution is an operation method that goes through Depthwise convolution and pointwise convolution as shown in Figure 9. It uses Depthwise convolution, which is a spatial operation, and pointwise convolution, which is a channel operation. To compare the general convolution, let us compare the convolution operation and the Depthwise Separable Convolution operation. First, check the operation process and computation amount of the convolution operation.

**Figure 10:**
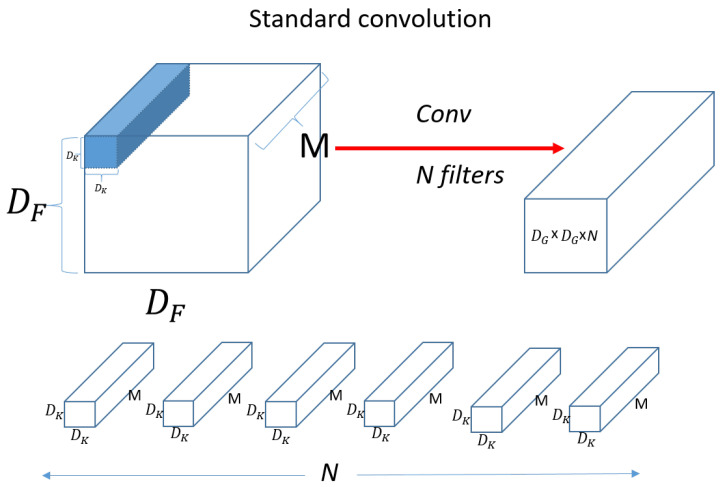
Standard convolution.

Figure 10 shows a standard convolution operation.

**Figure 11:**
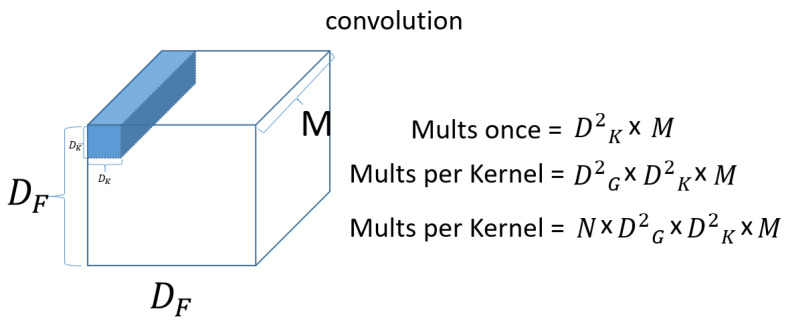
Convolution computation.

$D_{F}\times D_{F}\times M$ denotes input data as width x height x channel size, and M means channel. If the input is a three-dimensional color image, then M is 3 for R, G, and B. $D_{K}\times D_{K}\times M$ denotes the size of filter (kernel). Since the channel of the filter (kernel) is the same as the channel of the input, it will be the same as M. We then use N filters (kernels) to produce the output on the right, as shown in the figure. Figure 11 shows a standard convolution computation.

**Figure 12:**
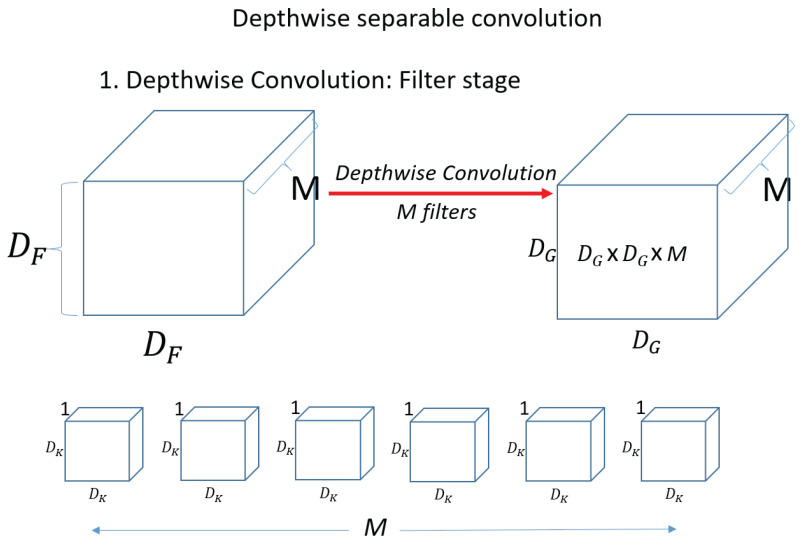
Depthwise convolution.

First, for a single kernel with one computation. $D_{k}\times D_{k}\times M=D_{k}^{2}\times M.\;$ Then, to process one set of input data, the total number of operations required is $D_{G}^{2}\times D_{K}^{2}\times M$. Because the kernel (filter) needs to move $D_{G}\times D_{G}=D_{G}^{2}$ for the input data. Then, since there are N filters (kernels), the output is $N\times \;D_{G}^{2}\times D_{K}^{2}\times M$. Next, check the Depthwise convolution operation, which is the first operation of the Depthwise Separable Convolution operation. Figure 12 shows a Depthwise convolution computation.

**Figure 13:**
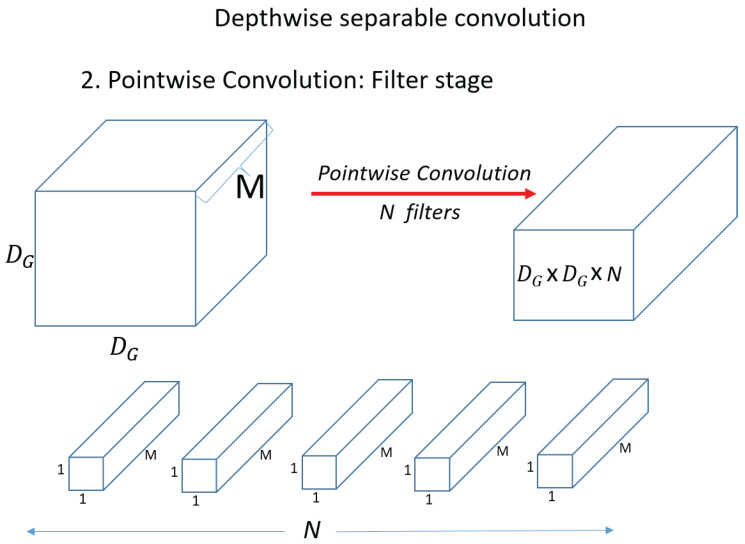
Pointwise convolution.

Unlike the above operation, in Depthwise convolution, the kernel (filter) has a channel of 1, and the operation is performed on only 1 channel of the input data. The shape of the kernel is $D_{k}\times D_{k}\times 1=D_{k}^{2}$. The amount of computation is $D_{k}^{2}$ when one kernel (filter) computes once on the input. Then, the operation is performed on one channel and we need $D_{G}^{2}\times D_{k}^{2}$. And since there are M kernels in total, the total computation is $M\times \;D_{G}^{2}\times \;D_{k}^{2}$. Figure 13 shows the pointwise convolution. In pointwise convolution, the kernel (filter) shape is 1×1×M When one kernel (filter) operates once on the input, it is $D_{G}\times D_{G}\times M$. Therefore, the total operation of pointwise convolution for N filters is equal to $N\times D_{G}^{2}\times M$. Therefore, the total operation of pointwise convolution for N filters is equal to $M\times D_{G}^{2}\times D_{k}^{2}+N\times G^{2}\times G^{2}\times M$. Compare standard convolution and Depthwise Separable Convolution and compare the amount of reduction compared to standard convolution.

(4) $$\frac{No.MultsinDepthwiseSeparableConv}{No.MultsinStandardConv}=\frac{\begin{array}{c} M\times D_{G}^{2}\left(D_{k}^{2}+N\right) \end{array}}{\begin{array}{c} N\times D_{G}\times D_{G}\times D_{k}\times D_{k}\times M \end{array}}$$

(5) $$\frac{No.MultsinDepthwiseSeparableConv}{No.MultsinStandardConv}=\frac{\begin{array}{c} D_{k}^{2}+N \end{array}}{\begin{array}{c} \left(D_{k}^{2}\times N\right) \end{array}}=\frac{1}{N}+\frac{1}{D_{k}^{2}}$$

(6) $$\frac{No.MultsinDepthwiseSeparableConv}{No.MultsinStandardConv}=\frac{\begin{array}{c} D_{k}^{2}+N \end{array}}{\begin{array}{c} \left(D_{k}^{2}\times N\right) \end{array}}=\frac{1}{N}+\frac{1}{D_{k}^{2}}=\frac{1}{1024}+\frac{1}{3^{2}}$$

Calculating Equations (4)–(6), it can be seen that the amount of calculation is reduced by about 1/9 times.

## 3. Few-Shot Learning Based Light-Weight WDCNN

### 3.1. Model Architecture

**Figure 14:**
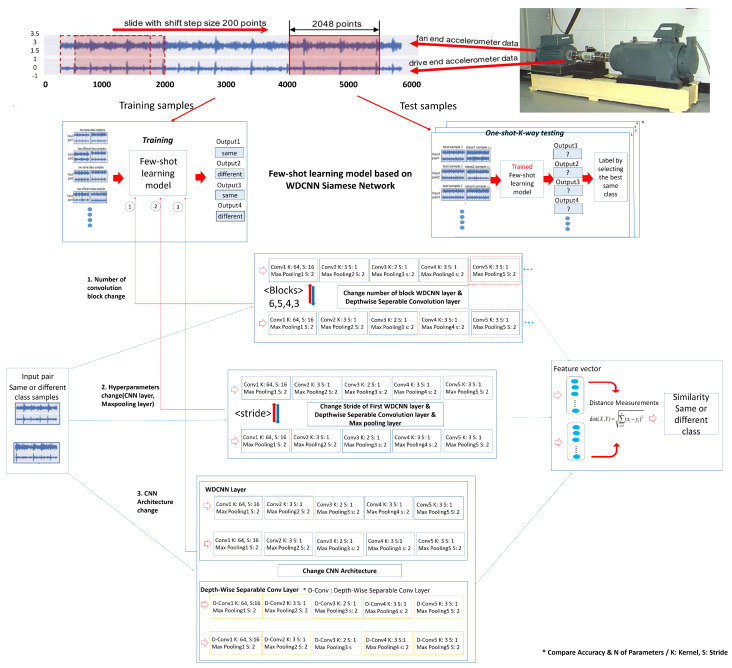
Model architecture.

Figure 14 shows the model architecture.

**Figure 15:**
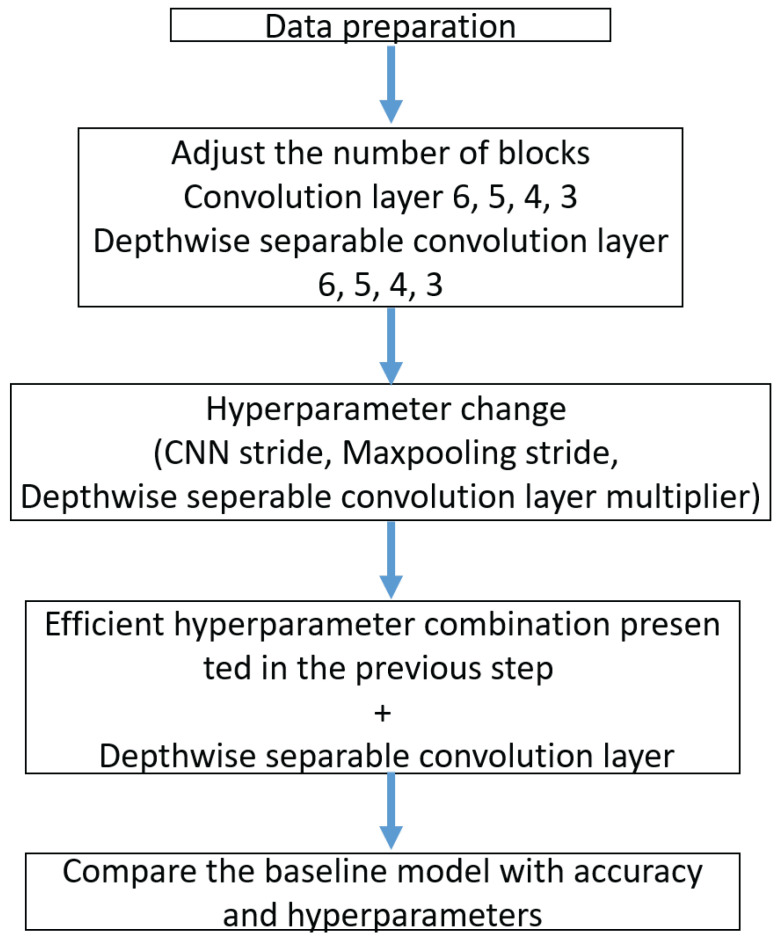
Model flow chart.

Figure 15 shows the model flow chart.

Figure 14 shows the three steps of the model architecture. First, we have the data preparation stage (Top), then the few-shot learning training & test (Middle), and finally the Siamese Network structure based on the WDCNN model (Bottom).

In the data preparation stage (Top), 12 k drive end bearing failure data from the Case Western Reserve University (CWRU) bearing data set were used as experimental data for performance verification. In the data preparation step, each sample is extracted from two vibration signals (ex: fan end, drive end). Half of the vibration signal is used to generate training samples and the other half is used to create test samples. The t raining samples are generated as a sliding window of size 2048 points, slid in 80-point shift steps. The test sample is also created without overlapping with the same window size.

The second stage is the few-shot model training (Middle). This training uses input data (two vibration signals) from a set of sample pairs of the same or different classes, which train the model to know the similarities and differences between the given data. Each neural network outputs two feature vectors extracted from two input images. The difference (distance) between two feature vectors after output distance metric = |h1 − h2|. After that, we use a dense layer to process the difference between the vectors. We apply the sigmoid activation function to obtain a number between 0 and 1. The similarity between the two images is measured, and if the two images are of the same class, the output is close to 1 and the other class is close to 0. The difference between the target value and the predicted scalar is measured using the loss function (cross-entropy). After measurement, Adam optimization is applied. As input, the loss is calculated by Equation (6) and the model is optimized by Equation (7).

### 3.2. Number of Convolution Block Change

Block 1 consists of a convolution layer and a pooling layer. Each model consists of six, five, four or three blocks. The accuracy and number of parameters of each model are compared. Table 2 represents the configuration in WDCNN. Five layers are the basic base model, experiment with four or three by reducing one block to No 9, 10 in Table 2, and add one block defined above after No 9, 10 for 6 layers. First, we experiment with six, five, four or three blocks of WDCNN composed of convolution layers. Table 2 shows the Structure of a few-shot learning model based on WDCNN.

Table 3 shows the structure of few-shot learning model based on Depthwise Separable Convolution layer WDCNN.

### 3.3. Hyperparameters Change (CNN Layer, Maxpooling Layer, Depthwise Separable Layer)

In Table 2 of the WDCNN model composed of convolution layers and Table 3 of the Depthwise Separable Convolution layer, hyperparameters are adjusted in five ways. As we adjust the hyperparameters in each method, we observe how the parameters and accuracy change. First, adjust the stride of the convolution layer in three ways. Adjust the stride to 23, 16, 9, 1 in the first convolution layer. Adjust the stride to 1, 2, 3, 4 in the second convolution layer. Adjust the stride 1, 2, 3, 4 in the last convolution layer. Second, adjust the first max pooling and last max pooling to 1, 2, 4. Third, adjust the depth multiplier hyperparameter to 1, 5, 10, 15 in the Depthwise Separable Convolution layer. Adjust the depth multiplier to 1, 5, 10, 15 on the first Depthwise Separable Convolution layer. Adjust the depth multiplier to 1, 5, 10, 15 on the second Depthwise Separable Convolution layer. Fourth, Add dropout and batch normalization to the baseline model. Fifth, Using the efficient hyperparameter combination of the experiment presented above and the Depthwise Separable Convolution layer, we compare the accuracy and hyperparameters with the baseline model.

## 4. Experiment and Results

### 4.1. Experiment Environments

Table 4 shows the experimental environment. The hardware used in this study consisted of an Intel Core i5-13600KF processor and Geforce RTX 4080. The CPU is made by intel and the GPU is made by nvidia. This equipment was supplied by Republic of Korea. The software uses Window 10, Tensorflow 2.10 and Python 3.9.

**Figure 16:**
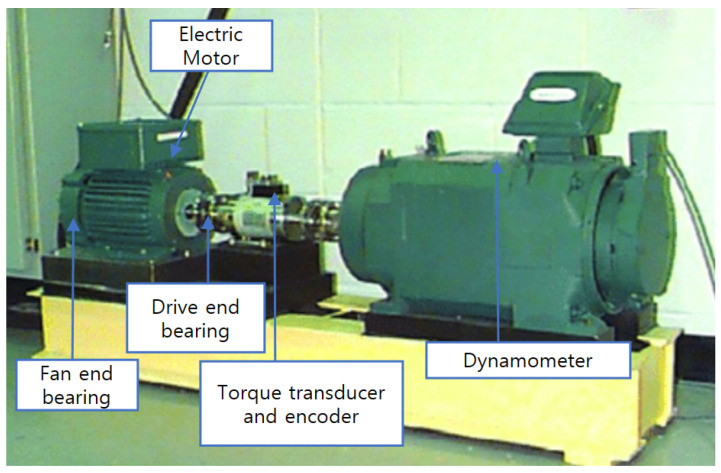
Bearing simulator.

The bearing data set used in this paper is from the Case Western Reserve University (CWRU), which consists of normal and defective bearings. In addition, it is a supervised learning-based dataset that labels normal and defective types. Defective types include inner race, outer race, and ball. The size of the defect is 0.007, 0.014, and 0.021. Data were collected on normal bearings and single-point drive end and fan end failures. The drive end was collected from samples measured at 12 k per second (12,000 vibration per second) and 48 k (48,000 vibration per second), and the fan end was collected from samples measured at 12 k per second (12,000 vibration per second). It was configured from 0 to 3 horsepower for each defect size. Outer race faults were measured for fault conditions at the 3, 6, and 12 o’clock positions. Figure 16 shows the bearing simulator of CWRU.

The CWRU simulator is composed of the dynamometer, electric motor, drive end bearing, fan end bearing, and torque transducer and encoder. Table 5 shows the description of rolling bearing datasets.

For the experimental data used in this paper, each sample was extracted from two vibration signals (fan, drive) as shown in Figure 16. The training and test samples are created with an 80-point sliding window that slides with a 2048-point shift step. Table 4 shows the description of rolling bearing datasets, with 10 types of defect labels, numbered 0 to 9, including normal labels. The data set consists of four parts A, B, C, and D. Loads 1, 2, and 3 correspond to data sets A, B, and C, respectively, and D is a data set combining loads 1, 2, and 3. In addition, data sets A, B, and C are composed of 660 training data and 25 test data, and data set D is a data set that combines the three working conditions A, B, and C, and consists of 1980 training sets and 75 test sets. In this experiment, 120, 200, 300, 600, 900, 1500, and 3000 training samples are randomly sampled from dataset D, respectively.

### 4.2. Evaluation Metric

Accuracy (%) is an index that determines how similar the predicted data are to the actual data. When accuracy is used as an evaluation metric, it is not a suitable evaluation metric when judging the performance of a machine learning model in an unbalanced label value distribution. Because it can distort performance. To overcome these limitations, it is used with various classification indicators. The equation for this parameter is as follows:

(7) $$Accuracy=\frac{\left|TP|+|TN\right|}{\left|TP|+|FP|+|FN|+|TN\right|}$$

Recall (%) is the ratio of what the model predicts to be true out of what is actually true. In other words, it is how much the model got right out of what was actually right. In statistics, it is used as sensitivity, and in other fields, it is also used as the term hit rate. The equation for this parameter is as follows:

(8) $$Recall\left(sensitivity\right)=\frac{\left|TP\right|}{\left|TP|+|FN\right|}$$

Precision (%) is the ratio of what the model says to be true to what is actually true. In other words, it measures how well the model correctly identifies what is actually true. The equation for this parameter is:

(9) $$Precision=\frac{\left|TP\right|}{\left|TP|+|FP\right|}$$

F1-score (%) is a method of expressing a single value through the harmonic average of two indicators (Precision (%), Recall (%)). It is used when the data label has an unbalanced structure or when the data between classification classes is severely imbalanced. The performance of the model can be accurately evaluated and the performance can be expressed as a single number. The equation for this parameter is:

(10) $$F1\text{-}Score=2*\frac{Precision*Recall}{Precision+Recall}$$

### 4.3. Number of Convolution Block Change Result

Table 6 shows the classification accuracy and the number of parameters for each block number of the WDCNN model composed of convolution layer.

In block five, the accuracy compared to the parameter is good in all samples. Therefore, block five is set as the baseline model during the hyperparameter experiment. Table 7 shows the classification accuracy and the number of parameters for each block number of the WDCNN model composed of Depthwise Separable Convolution layer.

**Figure 17:**
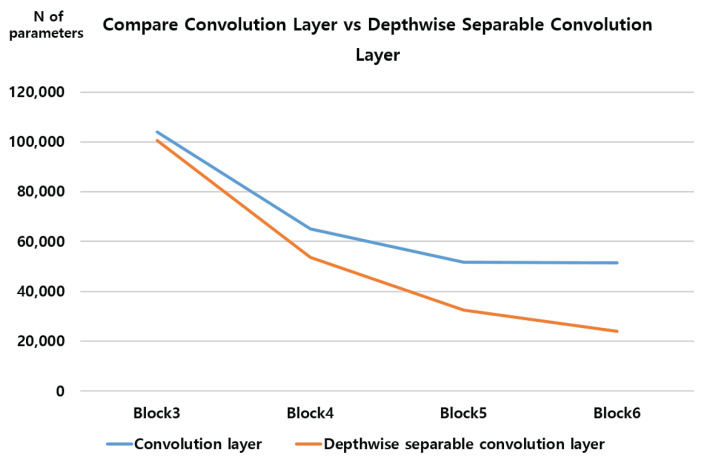
Number of parameters per block.

When adjusting the number of blocks with a Depthwise Separable Convolution layer, the parameter drops to 20,000 units in block six. However, it is not efficient because the accuracy compared to the parameters is very low. Figure 17 shows the change in the number of parameters as the number of blocks in each layer changes.

When the number of blocks increases from block three to block four, the number of parameters decreases rapidly. After that, in the convolution layer, the number of parameters decreases insignificantly, but in the Depthwise Separable Convolution layer, the number of parameters decreases slightly more rapidly than in the convolution layer. It can be seen that the Depthwise Separable Convolution layer has many fewer parameters than the convolution layer. In particular, in block six, the difference is more than doubled.

### 4.4. Hyperparameters Change (CNN Layer, Depthwise Separable Convolution Layer) Result

Adjust the first convolution layer stride to 23, 16, 9, 1. Table 8 shows the classification accuracy and number of parameters for each sample according to stride change in the first convolution layer of the WDCNN model composed of convolution layers.

The number of parameters decreases exponentially when the stride is reduced from 9 to 1 more than other stride changes. Table 9 shows number of parameters and accuracy per sample according to stride change difference.

Stride (23, 16) represents the difference between the sample accuracy and number of parameters obtained in stride 16 and the sample accuracy and number of parameters obtained in stride 23. Using the accuracy and the number of parameters obtained from Table 8, the difference between the accuracy and the number of parameters is calculated for each stride and sample in each step. Accuracy calculates the average of the accuracies after calculating the difference. Among the three stride (23, 16), stride (16, 9), and stride (9, 1), the part where the stride (9, 1) is calculated has the least increase in accuracy compared to the parameter. This is because the parameter increases exponentially from stride 1 to stride 9. Therefore, it can be seen that reducing the stride to 9 is most effective.

Adjust the second convolution layer stride to 1, 2, 3, 4. Table 10 shows the classification accuracy and number of parameters for each sample according to stride change in the second convolution layer of the WDCNN model composed of convolution layers.

When the stride was 3 or 4, the feature map became too small to perform the convolution or max pooling operation. so the experiment was performed only up to 1 and 2. Table 11 shows the number of parameters and accuracy per sample according to stride change difference.

When the parameter is reduced by 12,800, the accuracy decreases by 2.85% on average. Adjust the last convolution layer stride 1, 2, 3, 4. Table 12 shows the classification accuracy and number of parameters for each sample according to stride change in the last convolution layer of the WDCNN model composed of convolution layers.

Table 13 shows the number of parameters and accuracy per sample according to stride change difference.

Changes in strides 3 and 4 are not tested because the accuracy compared to the parameter is lower than reducing the stride of the second convolution layer.

Adjust the first max pooling to 1, 2, 4. Table 14 shows the classification accuracy and number of parameters for each sample according to stride change in the first max pooling of the WDCNN model composed of convolution layers.

Table 15 shows the number of parameters and accuracy per sample according to stride change difference.

When the first max pooling stride changes from 1 to 2, the parameter decreases to 19,301 and the accuracy increases by 0.15% on average. On the other hand, when increasing from 2 to 4, the parameter decreases by 12,699, but the accuracy decreases by about 5% on average, so the accuracy is significantly lower than that of the parameter.

Adjust last max pooling 1, 2, 4. Table 16 shows the classification accuracy and number of parameters for each sample according to stride change in the last max pooling of the WDCNN model composed of convolution layers.

Table 17 shows the number of parameters and accuracy per sample according to stride change difference.

Like the first maxpooling, the accuracy increases when the last maxpooling stride changes from 1 to 2, but the accuracy decreases when the stride increases from 2 to 4.

Adjust the depth multiplier hyperparameter to 1, 5, 10, and 15 in the first Depthwise Separable Convolution layer. Table 18 shows the classification accuracy and number of parameters for each sample according to depth multiplier change in the first Depthwise Separable Convolution layer of the WDCNN model composed of Depthwise Separable Convolution layer.

Table 19 shows number of parameters and accuracy per sample according to depth multiplier change difference.

From 1 to 10, the parameter accuracy increases efficiently, but from 10 to 15, the parameter accuracy does not increase efficiently.

Adjust the depth multiplier to 1, 5, 10, 15 on the second Depthwise Separable Convolution layer. Table 20 shows the classification accuracy and number of parameters for each sample according to depth multiplier change in the second Depthwise Separable Convolution layer of the WDCNN model composed of Depthwise Separable Convolution layer.

Table 21 shows the number of parameters and accuracy per sample according to depth_multiplier change difference.

Like the first Depthwise Separable Convolution layer From 1 to 10, the parameter accuracy increases efficiently, but from 10 to 15, the parameter accuracy does not increase efficiently.

Adjust the depth multiplier to 1, 5, and 10 on the last Depthwise Separable Convolution layer. Table 22 shows classification accuracy and the number of parameters for each sample according to depth multiplier change in the last Depthwise Separable Convolution layer of the WDCNN model composed of Depthwise Separable Convolution layer.

Table 23 shows the number of parameters and accuracy per sample according to depth multiplier change difference.

The first and second depth multipliers went up to 15, but the last depth multiplier only increased to 10 because the accuracy decreases whenever you increase to 1, 5, or 10.

Add dropout and batch normalization to the baseline model. Table 24 shows the classification accuracy for each sample according to the application of batch normalization and dropout to the WDCNN model.

Table 25 shows the number of parameters and accuracy per sample according to depth multiplier change difference.

When batch normalization is added to the baseline model, the accuracy compared to the parameter increases in most samples, but it drops significantly in sample 120. On the other hand, when dropout is added, the accuracy compared to the parameter increases in most samples without changing the parameter, and it does not decrease significantly in sample 120.

Create a Depthwise Separable Convolution layer with the hyperparameter combination presented above.

Table 26 shows the structure of the most efficient hyperparameter combination model (Depthwise Separable Convolution) for the method presented above.

Table 27 shows the accuracy results of proposed model.

Table 28 shows the F1-score results of proposed model.

Table 29 shows the number of parameters and accuracy (%) per sample according to model change difference.

In most samples, the accuracy of our proposed model is higher. However, the accuracy dropped in samples 120 and 200, with a significant decrease at 120.

## 5. Conclusions

In this study, we propose a light-weight WDCNN based on few-shot Learning to solve the problem of diagnosing bearing defects. While carrying out a bearing failure diagnosis study, we discovered the limitations of the existing research. First, it is practically difficult to obtain enough data samples to train all failure type classifications in actual manufacturing sites. Second, in previous studies, only accuracy was considered without considering parameters. To overcome these two limitations, we found an efficient combination that considers parameters and accuracy together with few-shot learning. With the method proposed in this paper, it is expected that the utilization of few-shot learning and future research will consider not only accuracy, but also parameters.

We implemented two strategies based on the Few-shot Learning-based Light-weight WDCNN Architecture and observed the changes in parameters and accuracy for each strategy. The first strategy adjusted the number of blocks to six, five, four, or three in the convolution layer and the Depthwise Separable Convolution layer. The second strategy adjusted the hyperparameters of the convolution layer and the Depthwise Separable Convolution layer in five ways. When adjusting the number of blocks in each layer, it was found that the accuracy compared to parameters was the most efficient at block five. Hyperparameters were adjusted in five ways. First, we adjusted the convolution layer stride. A stride of 9 was the best for the first convolution layer, 2 for the second layer, and 1 for the last layer. Second, 2 was the best when adjusting the first maxpooling and last maxpooling stride. Thirdly, when adjusting the depth multiplier of the Depthwise Separable Convolution, 10 was the best for the first and second Depthwise Separable Convolution layers. Fourth, we experimented by adding dropout and batch normalization to the baseline model. Finally, we experimented with a Depthwise Separable Convolution layer by combining efficient hyperparameters among the above methods. In most samples, we were able to maintain a high accuracy of about 90% or more compared to the parameter.

In the above experiment and the proposed model, the accuracy does not drop significantly when the parameters are reduced. However, when the number of samples is small (ex: 120, 200), the parameter is reduced, but the accuracy compared to the parameter is greatly reduced. Therefore, when the number of samples is larger, it shows higher performance with fewer parameters than the existing model.

Future research will proceed in three aspects. First, the CWRU dataset is composed of noise-free data. However, in the actual manufacturing site, the dataset contains noise. Therefore, it is necessary to experiment with data in the actual manufacturing site where the model of the hyperparameter combination presented is mixed with noise. Second, you can try other networks (ex: Matching network, Graph Neural network) that compensate for the shortcomings of the Siamese Network. Additional approaches to the distance-based approach could be researched. Third, in addition to the hyperparameter adjustment method presented in the paper above, it is possible to obtain fewer parameters and higher accuracy by adjusting the hyperparameters or combining models. 

## Figures and Tables

**Table 1:** Comparison between previous works and proposed methods.

	Dataset	Adjusting the Number of Blocks	Hyperparameter Tuning	Using Another Model	Learning with Less Data	Number of Parameters
Zhang et al. [27],	CWRU [31]	X	X	X	O	51,897
Han et al. [5], Oh et al. [6],	CWRU	X	X	O	X	over 51,897
Methods	CWRU	O	O	O	O	32,089

**Table 2:** Structure of few-shot learning model based on WDCNN.

No	Layer Type	Kernel Size/Stride	Kernel Number	Output Size (Width × Depth)	Padding
1	Convolution1	64 × 1/16 × 1	16	128 × 16	same
2	Max pooling1	2 × 1/2 × 1	16	64 × 16	valid
3	Convolution2	3 × 1/1 × 1	32	64 × 32	same
4	Max pooling2	2 × 1/2 × 1	32	32 × 32	valid
5	Convolution3	64 × 1/16 × 1	64	32 × 64	same
6	Max pooling3	2 × 1/2 × 1	64	16 × 64	valid
7	Convolution4	64 × 1/16 × 1	64	16 × 64	same
8	Max pooling4	2 × 1/2 × 1	64	16 × 64	valid
9	Convolution5	64 × 1/16 × 1	64	6 × 64	valid
10	Max pooling5	2 × 1/2 × 1	64	3 × 64	valid
11	Fully-connected	100	1	100 × 1	

**Table 3:** Structure of few-shot learning model based on Depthwise Separable Convolution layer WDCNN.

No	Layer Type	Kernel Size/Stride	Kernel Number	Output Size (Width × Depth)	Padding
1	Depthwise Separable Convolution1	64 × 1/16 × 1	16	128 × 16	same
2	Max pooling1	2 × 1/2 × 1	16	64 × 16	valid
3	Depthwise Separable Convolution2	3 × 1/1 × 1	32	64 × 32	same
4	Max pooling2	2 × 1/2 × 1	32	32 × 32	valid
5	Depthwise Separable Convolution3	64 × 1/16 × 1	64	32 × 64	same
6	Max pooling3	2 × 1/2 × 1	64	16 × 64	valid
7	Depthwise Separable Convolution4	64 × 1/16 × 1	64	16 × 64	same
8	Max pooling4	2 × 1/2 × 1	64	8 × 64	valid
9	Depthwise Separable Convolution5	64 × 1/16 × 1	64	6 × 64	valid
10	Max pooling5	2 × 1/2 × 1	64	3 × 64	valid
11	Fully-connected	100	1	100 × 1	

**Table 4:** Experiment Environments.

Hardware Environment	Software Environment
CPU: Intel Core TM i5-13600KF 3.50GHZ	Window 10
GPU: NVIDIA Geforce RTX 4080	Tensorflow 2.10, Python 3.9

**Table 5:** Description of rolling bearing datasets.

Fault	Location	None		Ball			Inner Race			Outer Race		Load
Fault Diameter (inch)		0	0.007	0.014	0.021	0.007	0.014	0.021	0.007	0.014	0.021	
Fault Labels		1	2	3	4	5	6	7	8	9	10	
Dataset A	Train	660	660	660	660	660	660	660	660	660	660	
	Test	25	25	25	25	25	25	25	25	25	25	1
Dataset B	Train	660	660	660	660	660	660	660	660	660	660	
	Test	25	25	25	25	25	25	25	25	25	25	2
Dataset C	Train	660	660	660	660	660	660	660	660	660	660	
	Test	25	25	25	25	25	25	25	25	25	25	3
Dataset D	Train	660	660	660	660	660	660	660	660	660	660	
	Test	75	75	75	75	75	75	75	75	75	75	1, 2, 3

**Table 6:** Classification accuracy (%) and number of parameters for each block number of the WDCNN model composed of a convolution layer.

	Sample	120	200	300	600	900	1500	3000	N of Parameters
Block									
3		82.53	89.83	89.47	93.43	96.40	98.91	99.33	103,993
4		90.91	94.65	93.74	95.95	97.80	98.97	99.35	65,145
5		92.43	94.87	94.05	96.84	98.19	98.95	99.43	51,897
6		89.77	93.38	91.79	96.52	96.83	98.70	99.28	51,449

**Table 7:** Classification accuracy (%) and number of parameters for each block number of the WDCNN model composed of Depthwise Separable Convolution layer.

	Sample	120	200	300	600	900	1500	3000	N of Parameters
Block									
3		74.22	78.92	82.69	87.18	89.55	93.11	96.48	99,145
4		76.33	82.28	84.87	87.61	92	94.23	97.05	52,297
5		77.80	82.35	85.61	91.28	93.27	95.95	98.04	31,049
6		75.14	82.02	84.88	90.38	93.00	95.67	97.97	22,601

**Table 8:** Classification accuracy (%) and number of parameters for each sample according to stride change in the first convolution layer of the WDCNN model composed of convolution layers.

	Sample	120	200	300	600	900	1500	3000	N of Parameters
Stride									
23		85.92	91.69	91.01	93.58	95.67	97.26	99.25	39,097
16		92.43	94.87	94.05	96.84	98.19	98.95	99.43	51,897
9		94.79	96.75	96.29	98.32	99.15	99.32	99.76	71,097
1		90.70	97.84	99.26	99.51	99.69	99.82	99.88	435,897

**Table 9:** Number of parameters and accuracy (%) per sample according to stride change difference.

Compared Strides	Sample 120	Sample 200	Sample 300	Sample 600	Sample 900	Sample 1500	Sample 3000	Average	N of Parameter
(23, 16)	6.50	3.17	3.03	3.26	2.51	1.68	0.17	2.90	12,800
(16, 9)	2.36	1.88	2.24	1.47	0.96	0.37	0.32	1.37	19,200
(9, 1)	−4.09	1.08	2.97	1.18	0.53	0.50	0.12	0.32	364,800

**Table 10:** Classification accuracy and number of parameters for each sample according to stride change in the second convolution layer of the WDCNN model composed of convolution layers.

	Sample	120	200	300	600	900	1500	3000	N of Parameters
Stride									
2		89.21	88.95	90.13	93.26	95.98	98.05	99.21	39,097
1		92.43	94.87	94.05	96.84	98.19	98.95	99.43	51,897

**Table 11:** Number of parameters and accuracy (%) per sample according to stride change difference.

Compared Strides	Sample 120	Sample 200	Sample 300	Sample 600	Sample 900	Sample 1500	Sample 3000	Average	N of Parameter
(2, 1)	−3.22	−5.91	−3.91	−3.58	−2.20	−0.89	−0.22	−2.85	12,800

**Table 12:** Classification accuracy (%) and number of parameters for each sample according to stride change in the last convolution layer of the WDCNN model composed of convolution layers.

	Sample	120	200	300	600	900	1500	3000	N of Parameters
Stride									
2		84.00	88.29	91.13	95.75	96.45	97.99	98.99	39,097
1		92.43	94.87	94.05	96.84	98.19	98.95	99.43	51,897

**Table 13:** Number of parameters and accuracy (%) per sample according to stride change difference.

Compared Strides	120	200	300	600	900	1500	3000	Average	N of Parameter
(2, 1)	−8.42	−6.57	−2.91	−1.09	−1.73	−0.95	−0.44	−3.16	12,800

**Table 14:** Classification accuracy (%) and the number of parameters for each sample according to stride change in the first max pooling of the WDCNN model composed of convolution layers.

	Sample	120	200	300	600	900	1500	3000	N of Parameters
Stride									
4		79.53	89.40	88.32	90.39	94.01	96.60	98.65	39,097
2		92.43	94.87	94.05	96.84	98.19	98.95	99.43	51,897
1		91.83	95.17	94.04	96.26	97.53	99.21	99.61	71,097

**Table 15:** Number of parameters and accuracy (%) per sample according to stride change difference.

Compared Strides	120	200	300	600	900	1500	3000	Average	N of Parameters
(4, 2)	−12.90	−5.46	−5.72	−6.44	−4.17	−2.34	−0.78	−5.40	12,800
(2, 1)	0.60	−0.30	0.01	0.58	0.65	−0.26	−0.18	0.15	19,200

**Table 16:** Classification accuracy (%) and number of parameters for each sample according to stride change in the last max pooling of the WDCNN model composed of convolution layers.

	Sample	120	200	300	600	900	1500	3000	N of Parameters
Stride									
4		91.33	94.76	92.28	96.10	97.54	98.68	99.33	45,497
2		92.43	94.87	94.05	96.84	98.19	98.95	99.43	51,897
1		91.85	94.39	94.99	96.19	97.67	98.97	99.43	64,697

**Table 17:** Number of parameters and accuracy (%) per sample according to stride change difference.

Compared Strides	120	200	300	600	900	1500	3000	Average	N of Parameter
(4, 2)	−1.09	−0.10	−1.76	−0.74	−0.64	−0.26	−0.10	−0.67	6400
(2, 1)	0.57	0.47	0.94	0.65	0.51	0.02	0.003	0.18	12,800

**Table 18:** Classification accuracy (%) and the number of parameters for each sample according to depth multiplier change in the first Depthwise Separable Convolution layer of the WDCNN model composed of Depthwise Separable Convolution layer.

	Sample	120	200	300	600	900	1500	3000	N of Parameters
Number									
15		76.59	82.25	84.97	91.09	93.07	96.73	98.71	33,289
10		77.46	81.50	83.92	91.29	93.06	96.14	98.20	32,489
5		75.46	82.55	84.44	89.63	92.366	95.40	98.08	31,689
1		72.90	78.92	81.72	89.01	90.23	94.07	96.91	31,049

**Table 19:** Number of parameters and accuracy (%) per sample according to depth multiplier change difference.

Compared Depth Multiplier	120	200	300	600	900	1500	3000	Average	N of Parameter
(15, 10)	−0.87	0.75	1.05	−0.2	0.01	0.59	0.51	0.26	800
(10, 5)	2.00	−1.04	−0.51	1.65	0.69	0.74	0.12	0.52	800
(5, 1)	2.56	3.62	2.71	0.62	2.13	1.33	1.16	2.02	640

**Table 20:** Classification accuracy (%) and number of parameters for each sample according to depth multiplier change in the second Depthwise Separable Convolution layer of the WDCNN model composed of Depthwise Separable Convolution layer.

	Sample	120	200	300	600	900	1500	3000	N of Parameters
Number									
15		78.70	82.15	84.49	90.76	92.89	95.67	97.32	38,889
10		77.05	82.62	85.44	89.46	92.32	95.47	97.47	36,089
5		74.11	78.80	83.38	89.88	92.41	95.01	97.21	33,289
1		72.90	78.92	81.72	89.01	90.23	94.07	96.91	31,049

**Table 21:** Number of parameters and accuracy (%) per sample according to depth multiplier change difference.

Compared Depth Multiplier	120	200	300	600	900	1500	3000	Average	N of Parameter
(15, 10)	1.65	−0.46	−0.94	1.30	0.56	0.20	−0.15	0.30	2800
(10, 5)	2.93	3.81	2.06	−0.41	−0.08	0.45	0.26	1.29	2800
(5, 1)	1.20	−0.12	1.65	0.86	2.18	0.94	0.30	1.00	2240

**Table 22:** Classification accuracy (%) and number of parameters for each sample according to depth multiplier change in the last Depthwise Separable Convolution layer of the WDCNN model composed of Depthwise Separable Convolution layer.

	Sample	120	200	300	600	900	1500	3000	N of Parameters
Number									
10		71.51	77.35	85.78	81.63	88.19	91.63	94.86	69,641
5		71.82	78.05	81.70	87.5	88.34	93.39	94.80	48,201
1		72.90	78.92	81.72	89.01	90.23	94.07	96.91	31,049

**Table 23:** Number of parameters and accuracy (%) per sample according to depth multiplier change difference.

Compared Depth Multiplier	120	200	300	600	900	1500	3000	Average	N of Parameter
(10, 5)	−0.31	−0.70	4.07	−5.89	−0.14	−1.75	0.05	−0.66	2800
(5, 1)	−1.07	−0.87	−0.01	−1.48	−1.88	−0.68	−2.11	−1.16	2240

**Table 24:** Classification accuracy (%) for each sample according to the application of batch normalization and dropout to the WDCNN model.

	Sample	120	200	300	600	900	1500	3000	N of Parameters
Model									
baseline		92.43	94.87	94.05	96.84	98.19	98.95	99.44	51,897
+batch normalization		84.06	94.71	97.51	99.16	99.40	99.66	99.68	52,857
+dropout		92.13	96.23	95.45	97.36	98.74	98.99	99.40	51,897

**Table 25:** Number of parameters and accuracy (%) per sample according to depth multiplier change difference.

Compared Model	120	200	300	600	900	1500	3000	Average	N of Parameters
(2, 1)	−8.36	−0.15	3.46	2.31	1.21	0.71	0.24	−0.08	960
(3, 1)	−0.30	1.36	1.40	0.51	0.55	0.043	−0.04	0.50	0

1. Baseline, 2. +Batch normalization, 3. +Dropout.

**Table 26:** Structure of few-shot learning model based on proposed model.

No	Layer Type	Kernel Size/Stride	Kernel Number	Output Size (Width × Depth)	Padding
1	Depthwise Separable Convolution	64 × 1/9 × 1	16	228 × 16	same
2	Batchnormalization		16	228 × 16	
3	dropout		16	228 × 16	
4	Max pooling1	2 × 1/2 × 1	16	114 × 16	valid
5	Depthwise Separable Convolution1	3 × 1/2 × 1	32	57 × 32	same
6	Batchnormalization		32	57 × 32	
7	dropout		32	57 × 32	
8	Max pooling2	2 × 1/2 × 1	32	28 × 32	valid
9	Depthwise Separable Convolution2	2 × 1/1 × 1	64	28 × 64	same
10	Batchnormalization		64	28 × 64	
11	dropout		64	28 × 64	
12	Max pooling3	2 × 1/2 × 1	64	14 × 64	valid
13	Depthwise Separable Convolution3	3 × 1/1 × 1	64	14 × 64	same
14	Batchnormalization		64	14 × 64	
15	dropout		64	14 × 64	
16	Max pooling4	2 × 1/2 × 1	64	7 × 64	valid
17	Depthwise Separable Convolution4	3 × 1/1 × 1	64	5 × 64	valid
18	Batchnormalization		64	5 × 64	
19	dropout		64	5 × 64	
20	Max pooling5	2 × 1/2 × 1	64	2 × 64	valid
21	Fully-connected	100	1	100 × 1	

**Table 27:** Classification accuracy (%) and number of parameters of baseline and proposed model.

	Sample	120	200	300	600	900	1500	3000	N of Parameters
Model									
baseline		92.43	94.87	94.05	96.84	98.19	98.95	99.43	51,897
proposed model		80.85	92.99	96.60	99.01	99.27	99.65	99.79	32,089

**Table 28:** Classification F1-score results of proposed model (%).

	Sample	120	200	300	600	900	1500	3000
Model								
proposed model		80.85	92.99	96.60	99.01	99.27	99.65	99.79

**Table 29:** Number of parameters and accuracy (%) per sample according to model change difference.

Compared Strides	120	200	300	600	900	1500	3000	Average	N of Parameters
(2, 1)	−11.57	−1.87	2.55	2.17	1.08	0.70	0.36	−0.94	19,808

1. baseline, 2. proposed model.

## Data Availability

The data used in the paper can be found at https://engineering.case.edu/bearingdatacenter (accessed on 23 June 2023). This is the dataset of a bearing research conducted by Case Western Reserve University.
